# Age and Overweight Are Not Contraindications for a Breast Reconstruction with a TMG-Flap—A Risk and Complication Analysis of a Retrospective Double Center Study Including 300 Patients

**DOI:** 10.3390/jcm10050926

**Published:** 2021-03-01

**Authors:** Karl Schwaiger, Laurenz Weitgasser, Maximilian Mahrhofer, Kathrin Bachleitner, Selim Abed, Julia Wimbauer, Elisabeth Russe, Thomas Schoeller, Gottfried Wechselberger

**Affiliations:** 1 Department of Plastic, Reconstructive and Aesthetic Surgery Hospital of the Brothers of St. John of God (Barmherzige Brüder), Paracelsus Medical University Salzburg, Kajetanerplatz 1, 5020 Salzburg, Austria; selim_abed@hotmail.com (S.A.); julia.wimbauer@bbsalz.at (J.W.); elisabeth.russe@bbsalz.at (E.R.); gottfried.wechselberger@bbsalz.at (G.W.); 2 Department of Plastic and Reconstructive Surgery, Marienhospital Stuttgart, Teaching Hospital of the Eberhard Karls University Tübingen, 72074 Tübingen, Germany; laurenz_weitgasser@hotmail.com (L.W.); max.mahrhofer@gmail.com (M.M.); kathrin.bachleitner@mail.de (K.B.); thomas.schoeller@vinzenz.de (T.S.)

**Keywords:** TMG flap, breast reconstruction, complication analysis, transverse myocutaneous gracilis flap, transverse upper gracilis flap, autologous breast reconstruction

## Abstract

Introduction: The transverse myocutaneous gracilis (TMG) flap has become a popular and reliable alternative for autologous breast reconstruction. Initially described as a valuable tissue source for women with low body-mass index, indications nowadays have widely expanded. The Western civilization demographic development with its aging population and the steady growing average BMI has led to increasing breast reconstructions with TMG flaps in overweight and aged individuals. Patients and Methods: A total of 300 TMG free flaps for unilateral autologous breast reconstruction were evaluated in the form of a retrospective double center cohort study. Data extraction, study group formation and statistical analysis (One-way analysis of variance (ANOVA), Pearson’s chi-squared statistical analysis and relative risk calculation) were done specifically to evaluate age and BMI as risk factors for postoperative complications and outcome. Results: No significant differences in patients’ age and BMI in the complication groups compared to the no-complication group could be found. No significant difference regarding the occurrence of complications could be found in any of the formed risk-groups. No significant increase of minor-, major- or overall complications, flap loss or revision surgeries were found in the elderly patient groups or for patients with overweight. Conclusion: Age and overweight do not significantly increase the risk for postoperative complications after breast reconstructions with free TMG flaps. The findings of this study support the fact that microsurgical breast reconstruction with a free TMG flap should not solely be reserved for younger patients and females with a lower BMI.

## 1. Introduction

Today, microsurgical free tissue transfer represents a standard tool of plastic surgeons for breast reconstruction around the globe. While free flaps from the lower abdomen, such as the deep inferior epigastric perforator (DIEP) flap, are widely respected as the gold standard and first-line flap for microsurgical breast reconstruction, numerous valuable other flaps represent important alternatives.

While sufficient abdominal fat tissue is a prerequisite for any abdominal-based tissue reconstruction, obesity and its comorbidities, such as diabetes or hypertension, are known risk factors for any surgical procedure, including microsurgery [1,2,3,4].

The thighs provide a popular alternative source for free flaps used for breast reconstruction offering numerous different angiosomes, which can be converted into various free flaps. A few years after its first description by Harii et al., the transverse musculocutaneous gracilis (TMG) flap became an increasingly popular alternative for free autologous tissue breast reconstruction [5,6,7]. Its consistent anatomy, excellent scar concealability and tissue quality make it a viable alternative in patients with abdominal scarring or lack of abdominal fat. Although there are plenty of scientific studies evaluating its safety and applicability, the TMG flap, in comparison to the DIEP flap, is still the second line tissue source for autologous breast reconstruction for most plastic surgeons [8,9,10].

Due to an exceedingly aging population and with obesity becoming one of the most common diseases in the industrialized world [11,12], we retrospectively reviewed the effects of advanced age and overweight in breast reconstruction with the TMG flap. As of yet, the TMG flap was only considered the first-line free flap in slim and athletic patients, who do not offer enough abdominal donor tissue. It can, however, serve as a reliable and valuable alternative flap for breast reconstruction in the overweight population as well. Average flap weight in slim patients can be between 200 and 300 g and can even be higher if the TMG flap is raised in an extended fashion [13]. However, even higher flap weights up to 400 g can be observed in some patients. Since abdominal flaps can be much larger in size in overweight patients, the TMG flap offers a useful alternative for microsurgical breast reconstruction in this patient population and should not only be reserved for patients with a lesser BMI.

Detailed risk stratification of the impact of higher age and overweight in patients having a breast reconstruction with TMG flaps has not been conducted to our knowledge so far. The aim of this study was to investigate potential risks and complications in a yet overlooked patient population receiving breast reconstructions with TMG flaps and identify a potential negative interrelation.

## 2. Materials and Methods

**Figure 1:**
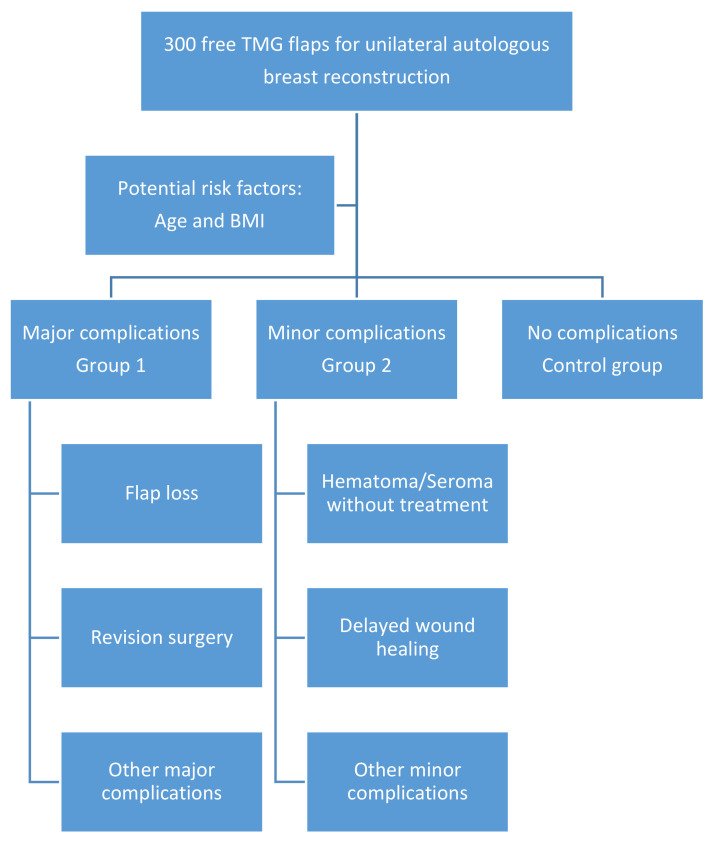
Schematic illustration of the study design.

Three hundred patients receiving a free TMG flap for unilateral breast reconstruction during the period of September 2010 to October 2020 were evaluated in the form of a retrospective double center cohort study (Figure 1). Data extraction and study group formation for this study was done specifically to evaluate age and BMI as risk factors for postoperative complications and outcome.

Upon analysis, 69 patients had a BMI higher or equal to 25 kg/m^2^, therefore falling into the overweight range according to the Center for Disease Control and Prevention Guidelines [14]. Ninety-five patients were older than 50, 26 patients older than 60. Patient characteristics were obtained from the medical records. Complications were specifically assessed separately in detail and classified according to the following categories: hematoma needing revision at the breast, hematoma treated conservatively, seroma breast, infection breast, wound healing disturbance breast, hematoma needing revision at the donor site, hematoma at the donor site treated conservatively, seroma at the donor site, wound healing disturbance at the donor site, contour deformity at the donor site, infection at the donor site, erythrocyte substitution, other complications. Additionally, all complications were categorized either as major or minor complications according to a slightly modified classification system of Neaman et al. [15] (Table 1).

All patients were treated according to a standardized two-team approach of simultaneous flap harvest and recipient site dissection. The optimized surgical technique has been published recently [16]. Both senior authors (Gottfried Wechselberger and Thomas Schoeller) have implemented identical pre-, intra- and postoperative protocols in each respective unit, thus offering a large group of patients without compromising the data set. Data were checked for consistency in terms of typing errors, and ranges were inspected for validity. One-way analysis of variance (ANOVA) was performed to evaluate the means of patients’ age and BMI in the groups “major complications”, “minor complications”, “flap loss,” and “revision surgery” (back to the operating room within 7 days after surgery) compared to “no complications” and “no flap loss”. Crosstabulation tables were formed for risk group evaluations according to the hypothesis (age and BMI as risk factors for complications) and were analyzed using two-tailed Pearson’s chi-squared statistical analysis. A value of *p* < 0.05 was considered statistically significant. Additionally, a specific risk group combining age and BMI (BMI over 25 kg/m^2^ and of older age) was built and compared to younger patients with lower BMI. The relative risks for the different groups for the occurrence of complication events were calculated. Homogeneity concerning additional well-known, relevant comorbidities (smoking, diabetes, radiation therapy) within the study groups (sorted according to BMI and age) was analyzed, and no significant difference could be found in any of the groups formed (*p* < 0.05). All statistical analyses in this report were performed by use of Social Science Statistics (socscistatistics.com, accessed on 20 December 2020) and GraphPad^®^ (GraphPad software, La Jolla, CA, USA). The study was conducted in accordance with the Ethical Principles for Medical Research involving Human Subjects of the Declaration of Helsinki.

## 3. Results

Patient characteristics are summarized in Table 2. ANOVA analysis showed no significant differences in patient age in the complication groups compared to the no complication group. The average age of the major complication group was 46.5 years (SD 11.5, *p* = 0.399), the average age of the minor complication group was 47.1 (SD 11.4, *p* = 0.679), and the average age of the overall complication group was 46.7 years (SD 11.3, *p* = 0.433) all compared to 47.7 years (SD 10) in the no complication group. ANOVA analysis also showed no significant differences in patients’ BMI of the groups. The average BMI of the major complication group was 22.8 kg/m^2^ (SD 2.9, *p* = 0.123), the average BMI of the minor complication group was 23.1 kg/m^2^ (SD 3.4, *p* = 0.287), and the average BMI of the overall complication group was 23.3 kg/m^2^ (SD 3.2, *p* = 0.143) all compared to 22.7 (SD 2.9) in the no complication group. The average age for patients who had a flap loss was 46.7 years (SD 9.1), and the average BMI was 23.9 kg/m^2^ (SD 3.13). No significant differences compared to the “no flap-loss” group were observed (*p*(*age*) = 0.819, *p*(*BMI*) = 0.170). Average age and BMI for the patients, who required a revision surgery was 46.35 kg/m^2^ (SD 12.36) years and 23.21 (SD 3.01), compared to 47.5 and 22.92 in the “no revision surgery” group ((*p*(*age*) = 0.426, *p*(*BMI*) = 0.480). The overall complication rate was calculated to be 49% (147/300). One hundred fifty-three patients (51.00%) had no complication, 83 patients (27.67%) had a major complication, and 70 patients (23.33%) had a minor complication (minor and major complications were assessed separately—double count possible). Flap loss was observed in 19 cases (6%). Revision surgery was required in 72 patients (24%). The average follow-up was 2.12 years.

**Figure 2:**
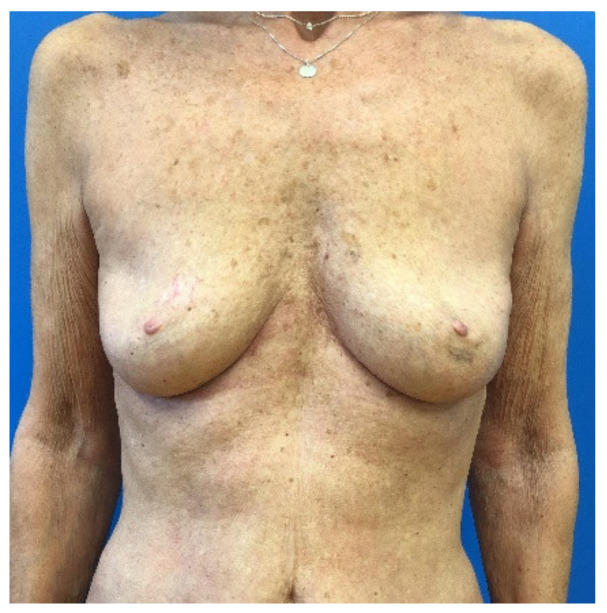
A 65-year-old patient presented to our department with the diagnosis of a multifocal invasive mamma carcinoma involving the nipple-areola complex. An interdisciplinary tumor-board decision was made to perform a subcutaneous mastectomy, including the nipple-areola complex and sentinel lymph node biopsy, plus immediate breast reconstruction with a TMG flap from the contralateral side.

**Figure 3:**
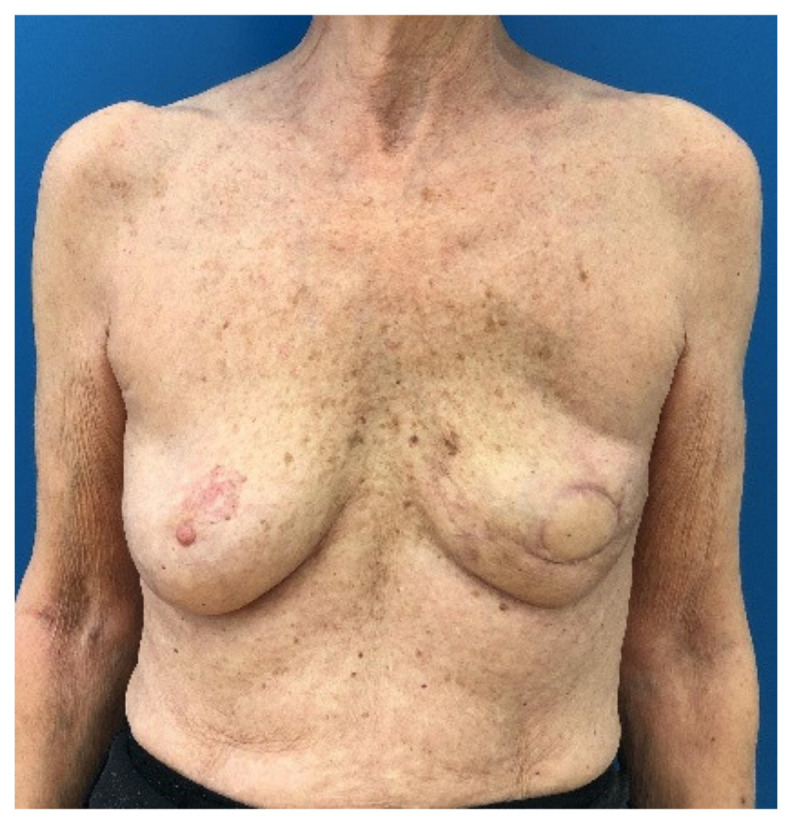
3 months postop picture. The patient denied further small esthetic corrections with lipofilling and nipple-areola reconstruction and was fully satisfied with the result.

**Figure 4:**
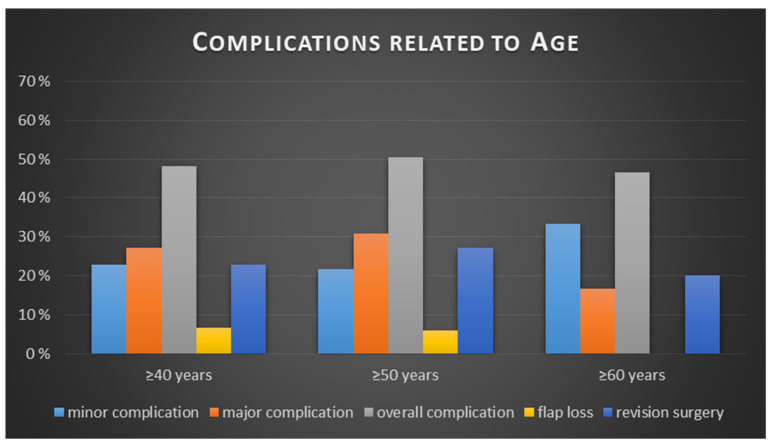
Relationship between age and the percentage of the different complications.

Crosstabulation tables were formed analyzing specific groups of risk according to the hypothesis. In general, 69 patients (23%) had a BMI higher or equal to 25 kg/m^2,^ and 133 patients (44.33%) were older than 50 years. Age groups were formed by sorting into over 40, over 50 and over 60 and compared to the younger patients regarding major complications, minor complications, overall complications, flap loss and revision surgery (Table 2). No significant difference regarding the occurrence of complications could be found in any of the formed age groups. No significantly higher rates of minor-, major- or overall complications, no significant more flap losses or revision surgeries were found in the elderly patient groups. Furthermore, relative risks (RR) were calculated and are also shown in Table 3. The highest relative risks were observed for revision surgery in patients over 50 (RR 1.26) and for minor complications for patients over 60 (RR 1.5) (nevertheless not significant, *p* = 0.267 and *p* = 0.408). For an example representing the elderly patient group, see Figure 2 and Figure 3, which show a 65-year-old patient treated with a TMG flap after breast cancer. Figure 4 shows the relationship between age and the percentage of the different complications. 

Complications were also categorized according to patient BMI (over and below BMI of 25 kg/m^2^) (Table 4). Regarding minor-, major-, and overall complications, mMinor complication rates, as well as for revision surgeries, no significant difference could be found within the groups. The highest relative risk calculated was 1.21 for major complications for patients with BMI over 25 (*p* = 0.460).

**Figure 5:**
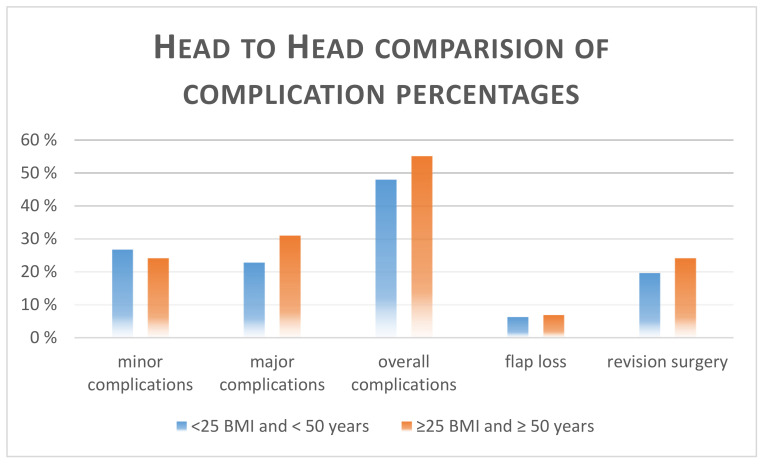
Schematic illustration of Table 4, comparison of patients with higher BMI and over 50 years to younger patients with lower BMI.

Table 5 and Figure 5 compare patients with higher BMI and over 50 years of age to younger patients with lower BMI. No significant difference was observed for the occurrence of the calculated complications. The highest relative risk calculation was observed for the major complications (RR 1.36, *p* = 0.349).

## 4. Discussion

Evaluation and assessment of potential risks and complications of microsurgical breast reconstruction procedures with free flaps have been conducted for over 20 years due to the high increase of these operations. Early on, being overweight or suffering from obesity, as well as older age, were identified as potential risk factors for complications. First, Chang et al. observed significantly higher rates of complications in overweight and obese patients having a breast reconstruction with free TRAM flaps [1]. Besides higher frequencies of lower abdominal bulging and herniation due to increased intraabdominal pressure in overweight patients [17,18], several studies confirmed a significantly higher overall risk for complications after reconstructions with abdominal based free flaps [3,4,19,20,21,22,23]. As an example, Boczar et al. [4] observed a reoperation rate of about 40% in obese patients and severe wound complications. Significantly high rates of donor site complication were observed in 16% of patients suffering from obesity. Only another study by Chang et al. [24] observed equal complication rates for the obese population in abdominally based free flaps. The studies cited reflect the majority of data found during our literature analysis concerning obesity or overweight as a risk factor for complications in microsurgical breast reconstruction.

A study published by Torabi et al. in 2018 found advanced age to be an independent risk factor after microsurgical breast reconstruction with DIEP flaps while also reporting disadvantages for wound healing and for flap loss in patients with higher age [25]. Contrariwise, Selber et al., Chang et al. and Oh et al. did not report significantly higher complication rates in elderly individuals receiving microsurgical breast reconstructions [26,27,28]. To our knowledge, all previous studies solely evaluated complication risk in advanced age patients after breast reconstruction with abdominally based free flaps (TRAM and DIEP) [25,26,27,28]. A thorough analysis of the literature could not reveal any present data of advanced age and its interrelation to complications after breast reconstructions with free TMG flaps.

About 20 years ago, the senior authors of this article introduced the TMG flap as a standardized and valuable free flap alternative for microvascular breast reconstruction [7,29]. A few years later, an additional paper with an emphasis on guidance for patient selection was published in order to reduce perioperative complications in breast reconstructions with TMG flaps [6]. In 2012 Locke et al. reported high complication rates and low patient and surgeon satisfaction in a series of 16 TMG flaps [30]. Four years later, Bodin et al. published an article about strategies to reduce the risk for complications when using the TMG flap for breast reconstruction [31]. A strict dissection of the exact TMG angiosome was recommended to avoid partial flap necrosis and donor site complications, such as hematoseroma and wound dehiscence. Another article by Nickl et al. published in 2018 reevaluated the surgical technique again and pointed out a modification to reduce complications further [32].

During our literature search, we were able to identify several studies, which evaluated a correlation of complications and advanced age in cosmetic thigh-lift surgeries, which in many ways represent a similar donor site wound as a free TMG flap does. In a study by Kühn et al. [33], age showed a statistically significant correlation to the occurrence of wound-associated problems (*p* = 0.02). The described mean difference of age in the described study, however, was only five years, which represents a limitation and likely points out a structural bias of the statistical analysis. Other studies (Nemerofsky et al. [34], Losco et al. [35], Arthrus et al. [36]) on cosmetic thigh- and body-lift operations were able to demonstrate a significant increase in complications with increased BMI (>28–32 kg/m^2^) but did do not observe any significant correlations of higher complications and age. However, these studies conclude that patients with a higher maximum body mass index before their massive weight loss had significant (*p* < 0.01) more complications. A direct comparison of the examined massive weight loss patient collective and patients receiving a breast reconstruction, who never had comparable massive weight alterations, is therefore conflicting and should be dismissed. The comparison of thigh lifts and free TMG flap donor sites are furthermore flawed by the fact that the evaluated thigh lifts were performed in a wise pattern fashion, while the free TMG flap donor sites were always closed in a horizontal thigh lift fashion. Therefore, although thigh lift surgeries are well-known to have high risks of postoperative hematoseromas, infections, wound healing disturbances and dehiscence, with an overall 68 percent of patients experiencing at least one complication [37], these complications cannot be expected in similarly high numbers after breast reconstructions with TMG flaps.

Although prior studies evaluated various risk factors for perioperative complications and were able to point out potential pitfalls and risks in a smaller patient sample size, a specific analysis of the risk stratification of age and increased BMI in breast reconstructions with the TMG flap has not been conducted so far. Our study group analyzed a larger-sized patient cohort of 300 TMG free flaps for breast reconstruction during the last 10 years and did an extensive evaluation to answer the question of any impact of age and overweight on complication risk. Our data analysis could not prove any significant relation of higher age and overweight to an increased rate of complications. The highest relative risks for revision surgery were observed in patients over 50 (RR 1.26) and for minor complications for patients over 60 (RR 1.5) (nevertheless not significant, *p* = 0.267 and *p* = 0.408). Regarding overweight, the highest relative risk calculated was 1.21 for major complications for patients with BMI over 25 kg/m^2^ (*p* = 0.460). We also formed a risk group, combining both risk factors according to the hypothesis and carried out a comparison against the most favorable patient group (<25 BMI (kg/m^2^) and < 50 years vs. ≥25 BMI (kg/m^2^) and ≥ 50 years). The highest relative risk calculation was observed for the major complications (RR 1.36, *p* = 0.349) in this comparison, but not no significant difference could be found.

Overall, our study demonstrates that advanced age and overweight do not play an essential role as a risk factor for complications in breast reconstruction with the free TMG flap in comparison to the majority of complication assessment studies for abdominally based free flaps. The study has several limitations, including its retrospective character, which increases the risk for observational bias. Although the present analysis was performed on the highest number of consecutive free TMG flaps ever evaluated in a single cohort study (*n* = 300), the overall number of patients over 70 years of age and with BMI higher than 30 (kg/m^2^) was relatively low for a profound statistical analysis. Thus, clear data on not only overweight (BMI >25 (kg/m^2^)) but obesity (BMI >30) could not be evaluated, and the highest age group was represented by sexagenarians only. Further studies with even larger sample sizes are needed to generate more powerful data in these patient groups. Furthermore, we need to point out the likely presence of a certain inclusion bias since the majority of patients qualifying for breast reconstruction with a TMG flap, in general, have a lower BMI compared to patients suitable for breast reconstruction with abdominal-based free flaps. Therefore, although the analyzed patient cohort is very large compared to most studies, the true amount of overweight patients assessed is not extensively high. With all limitations aside, we believe that this study underlines the important fact that microsurgical breast reconstructions with TMG flaps should not only be offered to younger and lower BMI patients but can safely be performed in patients with higher age and increased BMI. Although lower-abdominal-based free flaps, such as the DIEP flap, remain the gold standard in overweight and obese patients for autologous breast reconstruction, the free TMG flap offers a valuable and safe alternative when abdominal tissue is sparse, or an abdominal-based free flap is not feasible anymore.

## 5. Conclusions

Age and overweight do not significantly increase the risk for postoperative complications after breast reconstructions with free TMG flaps. The findings of this study support the fact that microsurgical breast reconstruction with a free TMG flap should not solely be reserved for younger patients and females with a lower BMI.

## Figures and Tables

**Table 1:** Classification of complications modified from Neaman et al. [15].

Major complications	1. Hematoma or flap insufficiency requiring surgical intervention
	2. Seroma requiring aspiration or surgery
	3. Wound-healing problems (also flap or fat necrosis) requiring surgery
	4. Infection requiring iv antibiotics
	5. Deep vein thrombosis/pulmonary embolism
Minor complications	1. Hematoma without treatment (+erythrocyte substitution with no other treatment necessary)
	2. Seroma without treatment
	3. Delayed wound healing
	4. Cellulitis (also fat necrosis) treated with oral antibiotics without hospitalization

**Table 2:** Patient characteristics.

Patient Characteristics	Number		%
Cases included	300		100
Age, years			
Median		48.0	
SD		10.6	
BMI, kg/m^2^			
Mean		22.9	
SD		3.0	
Radiotherapy			
Yes	116		38.7
No	183		61.0
Unknown	1		0.3
Hormone therapy			
Yes	120		40.0
No	180		60.0
Unknown	0		
Minor complications	70		23.3
Major complications	83		27.6
No complications	153		51.0
Revision needed	78		26.0

**Table 3:** Age groups were formed by sorting into over 40, over 50 and over 60 and compared to the younger patients regarding major complications, minor complications, overall complications, flap loss and revision surgery.

	<40 Years	≥40 Years	*p* Value
Minor complication	18 (25.00%)	52 (22.81%)	*p* = 0.644
No complication	35	118	
Relative risk for minor complication for ≥ 40: 0.91			
Major complication	21 (29.17%)	62 (27.19%)	*p* = 0.676
No complication	35	118	
Relative risk for major complication for ≥40: 0.93			
All complications	37 (51.39%)	110 (48.25%)	*p* = 0.642
No complication	35	118	
Relative risk for any complication for ≥ 40: 0.94			
Flap loss	4 (5.56%)	15 (6.58%)	*p* = 0.756
No flap loss	68	213	
Relative risk for flap loss for ≥40: 1.18			
Revision surgery	20 (27.78%)	52 (22.81%)	*p* = 0.389
No revision surgery	52	176	
Relative risk for revision surgery for ≥40: 0.82			
	**<50 years**	**≥50 years**	
Minor complication	41 (24.55%)	29 (21.80%)	*p* = 0.811
No complication	87	66	
Relative risk for minor complication for ≥ 50: 0.89			
Major complication	42 (25.15%)	41 (30.83%)	*p* = 0.356
No complication	87	66	
Relative risk for major complication for ≥ 50: 1.23			
All complications	80 (47.90%)	67 (50.38%)	*p* = 0.671
No complication	87	66	
Relative risk for any complication for ≥ 50: 1.05			
Flap loss	11 (6.59%)	8 (6.02%)	*p* = 0.839
No flap loss	156	125	
Relative risk for flap loss for ≥ 50: 0.91			
Revision surgery	36 (21.56%)	36 (27.07%)	*p* = 0.267
No revision surgery	131	97	
Relative risk for revision surgery for ≥50: 1.26			
	**<60 years**	**≥60 years**	
Minor complication	60 (22.22%)	10 (33.33%)	*p* = 0.408
No complication	137	16	
Relative risk for minor complication for ≥ 60: 1.50			
Major complication	78 (28.89%)	5 (16.67%)	*p* = 0.253
No complication	137	16	
Relative risk for major complication for ≥ 60: 0.58			
All complications	133 (49.26%)	14 (46.67%)	*p* = 0.788
No complication	137	16	
Relative risk for any complication for ≥ 60: 0.95			
Flap loss	19 (7.04%)	0 (0.00%)	*p* = 0.133
No flap loss	251	30	
Relative risk for flap loss for ≥ 60: 0.0			
Revision surgery	66 (24.44%)	6 (20.00%)	*p* = 0.589
No revision surgery	204	24	
Relative risk for revision surgery for ≥60: 0.82			

**Table 4:** Complications were also categorized according to patient BMI (over and below BMI of 25).

	<25 BMI (kg/m^2^)	≥25 BMI (kg/m^2^)	Total, *p* Value
Minor complication	56 (24.24%)	14 (20.29%)	*p* = 0.708
No complication	119	34	
Relative risk for minor complication for patients with BMI ≥ 25: 0.84			
Major complication	61 (26.41%)	22 (31.88%)	*p* = 0.460
No complication	119	34	
*Relative risk for major complication for patients with BMI ≥ 25: 1.21*			
All complications	112 (48.48%)	35 (50.72%)	*p* = 0.744
No complication	119	34	
Relative risk for any complication for patients with BMI ≥ 25: 1.05			
Flap loss	14 (6.06%)	5 (7.25%)	*p* = 0.723
No flap loss	217	64	
Relative risk for flap loss for patients with BMI ≥ 25: 1.20			
Revision surgery	54 (23.38%)	18 (26.09%)	*p* = 0.644
No revision surgery	177	51	
Relative risk for revision surgery for patients with BMI ≥ 25: 1.12			

**Table 5:** Comparison of patients with higher BMI and over 50 years to younger patients with lower BMI.

	<25 BMI (kg/m^2^) and < 50 Years	≥25 BMI (kg/m^2^) and ≥ 50 Years	Total, *p* Value
Minor complication	34 (26.77%)	7 (24.14%)	*p* = 0.931
No complication	66	13	
Relative risk for minor complication for patients with BMI ≥ 25 and over 50: 0.90			
Major complication	29 (22.83%)	9 (31.03%)	*p* = 0.349
No complication	66	13	
Relative risk for major complication for patients with BMI ≥ 25 and over 50: 1.36			
All complications	61 (48.03%)	16 (55.17%)	*p* = 0.488
No complication	66	13	
Relative risk for any complication for patients with BMI ≥ 25 and over 50: 1.15			
Flap loss	8 (6.30%)	2 (6.90%)	*p* = 0.906
No flap loss	119	27	
Relative risk for flap loss for patients with BMI ≥ 25 and over 50: 1.10			
Revision surgery	25 (19.69%)	7 (24.14%)	*p* = 0.592
No revision surgery	102	22	
Relative risk for revision surgery for patients with BMI ≥ 25 and over 50: 1.23			

## Data Availability

The data presented in this study are available on request from the corresponding author. The data are not publicly available due to ethical, legal and privacy issues.

## References

[1] Chang D.W., Wang B.-G., Robb G.L., Reece G.P., Miller M.J., Evans G.R.D., Langstein H.N., Kroll S.S. (2000). Effect of Obesity on Flap and Donor-Site Complications in Free Transverse Rectus Abdominis Myocutaneous Flap Breast Reconstruction. Plast. Reconstr. Surg..

[2] Kroll S.S., Netscher D.T. (1989). Complications of TRAM Flap Breast Reconstruction in Obese Patients. Plast. Reconstr. Surg..

[3] Seidenstuecker K., Munder B., Mahajan A.L., Richrath P., Behrendt P., Andree C. (2011). Morbidity of Microsurgical Breast Reconstruction in Patients with Comorbid Conditions. Plast. Reconstr. Surg..

[4] Boczar D., Huayllani M.T., Forte A.J., Rinker B. (2020). Microsurgical Breast Reconstruction in the Obese Patient Using Abdominal Flaps: Complication Profile and Patient Satisfaction. Ann. Plast. Surg..

[5] Harii K., Ohmori K., Sekiguchi J. (1976). The free musculocutaneous flap. Plast. Reconstr. Surg..

[6] Schoeller T., Huemer G.M., Wechselberger G. (2008). The Transverse Musculocutaneous Gracilis Flap for Breast Reconstruction: Guidelines for Flap and Patient Selection. Plast. Reconstr. Surg..

[7] Wechselberger G., Schoeller T. (2004). The Transverse Myocutaneous Gracilis Free Flap: A Valuable Tissue Source in Autologous Breast Reconstruction. Plast. Reconstr. Surg..

[8] Vega S.J., Sandeen S.N., Bossert R.P., Perrone A., Ortiz L., Herrera H. (2009). Gracilis Myocutaneous Free Flap in Autologous Breast Reconstruction. Plast. Reconstr. Surg..

[9] Craggs B., Vanmierlo B., Zeltzer A., Buyl R., Haentjens P., Hamdi M. (2014). Donor-Site Morbidity following Harvest of the Transverse Myocutaneous Gracilis Flap for Breast Reconstruction. Plast. Reconstr. Surg..

[10] Pülzl P., Schoeller T., Kleewein K., Wechselberger G. (2011). Donor-site morbidity of the transverse musculocutaneous gracilis flap in autologous breast reconstruction: Short-term and long-term results. Plast. Reconstr. Surg..

[11] Chooi Y.C., Ding C., Magkos F. (2019). The epidemiology of obesity. Metabolism.

[12] Beard J.R., Officer A., De Carvalho I.A., Sadana R., Pot A.M., Michel J.P., Lloyd-Sherlock P., Epping-Jordan J.E., Peeters G.G., Mahanani W.R. (2016). The World report on ageing and health: A policy framework for healthy ageing. Lancet.

[13] Wong C., Mojallal A., Bailey S.H., Trussler A., Saint-Cyr M. (2011). The extended transverse musculocutaneous gracilis flap: Vascular anatomy and clinical implications. Ann. Plast. Surg..

[14] Defining Adult Overweight and Obesity. https://www.cdc.gov/obesity/adult/defining.html.

[15] Neaman K.C., Armstrong S.D., Baca M.E., Albert M., Vander Woude D.L., Renucci J.D. (2013). Outcomes of traditional cosmetic abdominoplasty in a community setting: A retrospective analysis of 1008 patients. Plast. Reconstr. Surg. März..

[16] Wechselberger G., Schwaiger K. (2020). Transverse Upper Gracilis Flap in Breast Reconstruction. Breast Reconstruction.

[17] Nahabedian M.Y., Momen B. (2005). Lower Abdominal Bulge after Deep Inferior Epigastric Perforator Flap (DIEP) Breast Reconstruction. Ann. Plast. Surg..

[18] Blondeel P., Boeckx W., Vanderstraeten G., Lysens R., Van Landuyt K., Tonnard P., Monstrey S., Matton G. (1997). The fate of the oblique abdominal muscles after free TRAM flap surgery. Br. J. Plast. Surg..

[19] Moran S.L., Serletti J.M. (2001). Outcome comparison between free and pedicled TRAM flap breast reconstruction in the obese patient. Plast. Reconstr. Surg..

[20] Greco J.A., Castaldo E.T., Nanney L.B., Wu Y.C., Donahue R., Wendel J.J., Hagan K.F., Shack R.B. (2008). Autologous breast reconstruction: The Vanderbilt experience (1998 to 2005) of independent predictors of displeasing outcomes. J. Am. Coll. Surg..

[21] Ochoa O., Chrysopoulo M., Nastala C., Ledoux P., Pisano S. (2012). Abdominal wall stability and flap complications after deep inferior epigastric perforator flap breast reconstruction: Does body mass index make a difference? Analysis of 418 patients and 639 flaps. Plast. Reconstr. Surg..

[22] Lee K.-T., Mun G.-H. (2016). Effects of Obesity on Postoperative Complications After Breast Reconstruction Using Free Muscle-Sparing Transverse Rectus Abdominis Myocutaneous, Deep Inferior Epigastric Perforator, and Superficial Inferior Epigastric Artery Flap. Ann. Plast. Surg..

[23] Palve J.S., Luukkaala T.H., Kääriäinen M.T. (2020). Predictive risk factors of complications in different breast reconstruction methods. Breast Cancer Res. Treat..

[24] Chang E.I., Liu J. (2018). Prospective Evaluation of Obese Patients Undergoing Autologous Abdominal Free Flap Breast Reconstruction. Plast. Reconstr. Surg..

[25] Torabi R., Stalder M.W., Tessler O., Bartow M.J., Lam J., Patterson C., Wise M.W., Dupin C.L., Hilaire H.S. (2018). Assessing Age as a Risk Factor for Complications in Autologous Breast Reconstruction. Plast. Reconstr. Surg..

[26] Selber J.C., Bergey M., Sonnad S.S., Kovach S., Wu L., Serletti J.M. (2009). Free Flap Breast Reconstruction in Advanced Age: Is It Safe?. Plast. Reconstr. Surg..

[27] Chang E.I., Vaca L., DaLio A.L., Festekjian J.H., Crisera C.A. (2011). Assessment of Advanced Age as a Risk Factor in Microvascular Breast Reconstruction. Ann. Plast. Surg..

[28] Oh D., Flitcroft K., Brennan M., Spillane A. (2016). Patterns and outcomes of breast reconstruction in older women—A systematic review of the literature. Eur. J. Surg. Oncol. (EJSO).

[29] Wechselberger G., Schoeller T., Bauer T., Schwabegger A., Ninkovic M., Rainer C., Ninkovic M. (2001). Surgical technique and clinical application of the transverse gracilismyocutaneous free flap. Br. J. Plast. Surg..

[30] Locke M.B., Zhong T., Mureau M.A., Hofer S.O. (2012). Tug ‘O’ war: Challenges of transverse upper gracilis (TUG) myocutaneous free flap breast reconstruction. J. Plast. Reconstr. Aesthetic Surg..

[31] Bodin F., Dissaux C., Dupret-Bories A., Schohn T., Fiquet C., Bruant-Rodier C. (2015). The transverse musculo-cutaneous gracilis flap for breast reconstruction: How to avoid complications. Microsurgery.

[32] Nickl S., Nedomansky J., Radtke C., Haslik W., Schroegendorfer K.F. (2018). Optimization of breast reconstruction results using TMG flap in 30 cases: Evaluation of several refinements addressing flap design, shaping techniques, and reduction of donor site morbidity. Microsurgery.

[33] Kuhn S., Klettenheimer A., Küenzlen L., Kiehlmann M., Schlosshauer T., Djedovic G., Rieger U.M. (2019). Outcome, complications, and body mass index correlation of horizontal and combined horizontal and vertical thigh lift: A 16-year single-center experience. J. Cutan. Aesthetic Surg..

[34] Nemerofsky R.B., Oliak D.A., Capella J.F. (2006). Body Lift: An Account of 200 Consecutive Cases in the Massive Weight Loss Patient. Plast. Reconstr. Surg..

[35] Losco L., Roxo A.C., Roxo C.W., Torto F.L., Bolletta A., De Sire A., Aksoyler D., Ribuffo D., Cigna E. (2019). Lower Body Lift After Bariatric Surgery: 323 Consecutive Cases Over 10-Year Experience. Aesthetic Plast. Surg..

[36] Arthurs Z.M., Cuadrado D., Sohn V., Wolcott K., Lesperance K., Carter P., Sebesta J. (2007). Post-bariatric panniculectomy: Pre-panniculectomy body mass index impacts the complication profile. Am. J. Surg..

[37] Oranges C.M., Sisti A. (2015). Medial Thigh Lift in the Massive Weight Loss Population: Outcomes and Complications. Plast. Reconstr. Surg..

